# Canaloplasty and trabeculotomy with the OMNI^®^ surgical system in OAG with prior trabecular microbypass stenting

**DOI:** 10.1007/s10792-022-02553-6

**Published:** 2022-10-13

**Authors:** Daniel C. Terveen, Steven R. Sarkisian, Steven D. Vold, Deepan Selvadurai, Blake K. Williamson, Deborah G. Ristvedt, Adam R. Bleeker, Kavita Dhamdhere, Jaime E. Dickerson

**Affiliations:** 1 Vance Thompson Vision, Sioux Falls, SD USA; 2 Oklahoma Eye Surgeons, PLLC., Oklahoma City, OK USA; 3 Vold Vision, Fayetteville, AR USA; 4 Buffalo Ophthalmology, Williamsville, NY USA; 5 The Williamson Eye Center., Baton Rouge, LA USA; 6 Vance Thompson Vision, Alexandria, MN USA; 7 University of South Dakota Sanford School of Medicine, Sioux Falls, SD USA; 8 Sight Sciences, Inc., Menlo Park, CA USA; 9 Mahatma Gandhi Medical College and Research Center, Wardha, India; 10 North Texas Eye Research Institute, University of North Texas Health Science Center, Fort Worth, TX USA

**Keywords:** Open-angle glaucoma, Trabeculotomy, Viscodilation, OMNI surgical system, iStent, MIGS

## Abstract

**Purpose:**

Evaluate effectiveness and safety outcomes for patients treated with canaloplasty and trabeculotomy previously treated with a trabecular microbypass stent (TBS).

**Methods:**

Retrospective, multicenter, IRB approved study. Patients treated with TBS (iStent/iStent inject, Glaukos) and subsequently with OMNI surgical system (OSS) (Sight Sciences). From 5 practices in 5 US states. Open-angle glaucoma (OAG), minimum 3 months follow-up after OSS surgery, Pre-OSS IOP ≥ 17 mmHg on ≥ 1 medication. No glaucoma procedures between TBS and OSS. Endpoints: proportion with ≥ 20% reduction in IOP, IOP between 6 and 18 mmHg, mean IOP, change in IOP, mean number of medications. Adverse events and secondary surgical interventions (SSI). Mann–Whitney rank sum test compared pre-OSS IOP and medications with follow-up.

**Results:**

Twenty seven patients. Average age (SD) 72.2 (10.8), 22/27 primary OAG (82%), mean MD − 6.2 (7.0) dB. Mean IOP before OSS 22.3 (4.3) mmHg on 2.2 (1.3) medications. At last follow-up (mean 11 months) IOP was 17.2 mmHg on 1.8 medications, − 5.1 mmHg (− 23%, *p* < .001), − 0.4 meds (− 18%, *p* = .193); ≥ 20% IOP reduction (41%), IOP ≤ 18 (56%). Adverse events were non-serious. Hyphema > 1 mm (3, 11%), BCVA decrease (4, 15%), IOP spike (2, 7%). SSI (4, 15%) had higher pre-OSS IOP (23.4 mmHg) and worse MD (− 9.6 dB).

**Conclusion:**

Patients uncontrolled by medication and a prior TBS would once have been candidates for trabeculectomy and tube shunts. OSS offered a minimally invasive option that provided IOP control and avoidance of traditional surgery for the majority over follow-up averaging 11 months and up to 42 months.

## Introduction

Minimally invasive glaucoma surgery (MIGS) has changed the treatment of mild to moderate open-angle glaucoma dramatically over a relatively short span of time [1, 2]. The favorable safety profile, particularly in conjunction with cataract surgery, has resulted in a shift to surgical intervention in earlier disease, with the goal of delaying or obviating the need for traditional filtration surgery [3, 4].

MIGS have been a part of the surgical toolbox for several years; the Trabectome (NeoMedix, Tustin, CA) was cleared in 2006 presaging a completely new category of glaucoma surgery termed MIGS in 2008 [5]. Since that time a plethora of MIGS devices and procedures have been developed and are currently available. However, consensus regarding which MIGS to use and where specific MIGS technologies fit within the glaucoma treatment algorithm remains elusive in part due to the paucity of head-to-head clinical trials [4–6].

Some MIGS, specifically implantable microstents, are labeled for use in mild-moderate glaucoma at the time of cataract surgery. For patients with early glaucoma, cataract surgeons may implant a trabecular bypass stent (TBS) in the same operative session as cataract surgery knowing that they add little risk to the operation and provide some IOP lowering benefit beyond that obtained by lens extraction alone [7]. Glaucoma is a progressive disease and one that often progresses despite treatment. The aim of the present study is to assess the effectiveness of a second, non-implant, MIGS intervention, canaloplasty and trabeculotomy (OMNI Surgical System or OSS), in eyes that are no longer adequately controlled by prior implantation with a trabecular microbypass stent and medications.

## Methods

TREY was a multicenter, retrospective, observational, consecutive study of all eyes meeting eligibility criteria treated with the OMNI system from five multi-subspecialty ophthalmic practices in five US states (AR, LA, NY, OK, SD). Eligible patients were 18 years of age or more, had a diagnosis of open-angle glaucoma (OAG) including pigmentary and pseudoexfoliative glaucomas, were treated with a trabecular bypass stent (TBS) (iStent or iStent inject, Glaukos, San Clemente, CA, USA) and subsequently with the OMNI surgical system (OSS) (Sight Sciences Inc., Menlo Park, CA, USA), and had a pre-OSS baseline (BL) IOP of at least 17 mmHg and were under treatment with at least 1 ocular hypotensive medication. TBS surgeries were between September 6, 2012 and December 9, 2020; OSS surgeries were from July 11, 2018 to August 11, 2021. Patients were excluded if there had been any surgical or laser treatment for their glaucoma between the TBS implantation and the OSS surgery including laser trabeculoplasty, cyclodestructive/ciliary ablation procedures, any other MIGS, or traditional (i.e., trabeculectomy or tube shunt) glaucoma surgery.

A minimum of 3 months of follow-up from the date of surgery with the OSS was required. Exceptions were allowed for patients if they had required a secondary surgical or laser procedure for IOP control (SSI) prior to 3 months. Only one eye per patient was enrolled although the study protocol allowed both eyes of a patient to be enrolled if both met eligibility criteria.

Medical records were reviewed for demographic and medical history information, preoperative and postoperative IOP, medication use data, best corrected visual acuity (BCVA), adverse events, and any SSI. Date of TBS implantation and number of ophthalmic follow-up visits between TBS and OSS were also collected. Surgical information including clock hours of canaloplasty and trabeculotomy, and any intra-operative complications were abstracted from the operative notes. Follow-up data (post-OSS) was collected at month 1 (15 to 60 days), month 3 (90 to 122 days), and last available follow-up (> 122 days). All patients had TBS implantation in conjunction with phacoemulsification cataract surgery, and therefore all eyes were pseudophakic at the time of the OSS procedure. In 12 (45%) of eyes the TBS was removed at the time of OSS; in the remaining eyes the OSS procedure was readily carried out without interference from the single stent by accessing the canal to either side.

The study was reviewed by the Institutional Review Board (WCG IRB, Puyallup, WA, USA; IRB Protocol No: 20213289) and waiver of consent was granted due to the retrospective non-interventional nature of the study. All patient data was treated with confidentiality, in accordance with the Declaration of Helsinki. This study is not considered an “Applicable Clinical Trial” under 42 CFR 11.22(b) and is exempt from listing on clinicaltrials.gov.

All subjects had undergone a complete ophthalmic examination including slit-lamp and dilated fundus examinations, Goldmann applanation tonometry, gonioscopy, and automated perimetry prior to surgery. As this study is a retrospective chart review and all ophthalmic exams were carried out as part of the surgeons normal standard practice, standardization of methodology (e.g., operator/reader IOP measurements, standard perimetry program) was not possible. The preoperative (OSS) exam was a median of 20.5 days prior to surgery (mean 38.5 days, 90th percentile 58.7 days). The IOP measured and the number of IOP-lowering medications at this exam were used as the BL IOP and BL number of medications.

### Surgical technique

Surgical technique for performing canaloplasty and trabeculotomy with the OSS has been previously described [8]. Briefly, and in general, a small (~ 2 mm) temporal clear corneal incision was created. Following irrigation of the anterior chamber (AC) with 2% lidocaine and deepening with viscoelastic, the head was tilted away from the surgeon and the microscope was tilted toward the surgeon for gonioscopic visualization (generally 30–40 degrees head; 30–40 degrees microscope). In approximately half (45%) of eyes the TBS was removed using forceps without complication before the OSS procedure. The OSS was introduced through the incision into the AC and was advanced across the AC, positioned nasally at the desired location and a small < 1 mm goniotomy was created with the cannula tip. The microcatheter was then advanced into Schlemm's canal for 180°. Withdrawal of the microcatheter using the thumb wheel deposited a controlled amount of viscoelastic viscodilating the canal and distal outflow pathway. Readvancement of the microcatheter to the desired extent and subsequent withdrawal by removal of the OSS through the corneal incision unroofed Schlemm’s canal. To treat the other hemisphere the OSS was rotated 180 degrees and reinserted into the eye repeating the steps outlined above. On completion of the procedure the AC was irrigated with BSS to remove viscoeleastic, and the chamber was pressurized. With minor inter-surgeon variations, a standard postoperative regimen of topical steroid, and antibiotic was prescribed. Both hemispheres were treated in all cases.

### Outcome measures

Primary effectiveness was the proportion of patients with a ≥ 20% reduction in IOP from the pre-OSS BL with no additional laser or surgical intervention at the last follow-up assessment. Secondary effectiveness included the proportion of patients with IOP between 6 and 18 mmHg (inclusive), percentage change in IOP at last follow-up from pre-OSS BL, mean IOP at last follow-up, mean number of ocular hypotensive medications at last follow-up, and cumulative probability of survival (no SSI). Adverse events, and SSI are reported.

### Statistical analysis

The sample size for this descriptive study was a “convenience sample” that included all eligible patients. There was no a priori hypothesis and sample size was not set according to statistical power considerations. The Full Analysis Set (FAS) included all patients meeting all inclusion criteria, and no exclusion criteria. Patients that underwent an SSI prior to the endpoint were considered to be failures in binary endpoint analyses (e.g., proportion with a ≥ 20% reduction in IOP) and IOP or medication use data subsequent to the SSI were excluded due to the confounding influence of the secondary procedure. The safety analysis set included all enrolled subjects.

Demographics and BL characteristics were analyzed with descriptive statistics (mean, maximum, minimum, standard deviation). The proportions for the primary binary outcome and corresponding standard errors and confidence bounds were calculated. The mean IOP and the number of ocular hypotensive medications is reported at each post-OSS time and compared to BL using the nonparametric Mann–Whitney rank sum test. Missing data due to a procedure not being done or a missed visit were treated as missing. No imputation was carried out.

Adverse events (AEs) were classified as intraoperative or postoperative as well as serious or non-serious. The number and the percent of eyes reporting at least 1 adverse event of a given type are summarized. The number and percent of eyes reporting with BCVA of 20/20 or better, 20/25 or better, 20/32 or better, 20/40 or better, worse than 20/40 to 20/80, worse than 20/80 to 20/200, and worse than 20/200 at each visit are summarized. The number and percent of eyes with an SSI is also presented.

## Results

Twenty-seven eyes meeting eligibility criteria were enrolled. Mean duration between TBS implantation and OSS was 4.6 years (median 5.1 years, maximum 8.5 years, minimum 6 months). 26 of 27 patients had been implanted with the 1st generation iStent (and in one of these, three stents), 1 with an iStent inject. Mean follow-up after OSS was 11.0 months (minimum 3 months, quartile 1 3.7 months, median 6.6 months, quartile 3 12.3 months, maximum 41.2 months,). Most patients were White (96%), had a diagnosis of primary open-angle glaucoma (POAG, 81.5%) with genders equally represented (13 female, 14 male). Average (SD) age at the time of OSS surgery was 72.2 (10.8) years. Most eyes had either moderate (− 5.01 to − 12.00 dB MD; 8, 30%) or advanced (− 12.01 to − 20.00 dB MD; 7, 26%) glaucoma. Demographic and baseline characteristics are presented in Table 1.

**Table 1:** Demographic and baseline characteristics

Parameter	Value (*N* = 27)
Age (yr), mean (SD)	72.2 (10.8)
*Gender, n (%)*	
Female	13 (48)
Male	14 (52)
*Race/Ethnicity, n (%)*	
White	26 (96)
Black/African-American	1 (4)
*Glaucoma Diagnosis n, (%)*	
Primary open-angle	22 (81.5)
Pseudoexfoliation	3 (11.1)
Pigmentary	2 (7.4)
Visual Field Mean Deviation (dB), mean (SD)	− 6.2 (7.0)
*Stage n, (%)*	
Mild (MD > − 5.01)	12 (44.4)
Moderate (MD ≤ − 5.01, > − 12.01)	8 (29.6)
Advanced (MD ≤ − 12.01)	7 (25.9)
Cup to Disc ratio (SD)	0.7 (0.2)
*Pre-operative (OSS) IOP (mmHg), mean (SD)*	
ALL	22.3 (4.3)
POAG (*n* = 22)	21.7 (3.2)
PXG (*n* = 3) and PG (*n* = 2)	24.7 (7.6)
*Pre-operative (OSS) Medications, mean (SD)*	
ALL	2.2 (1.3)
POAG (*n* = 22)	2.4 (1.3)
PXG (*n* = 3) and PG (*n* = 2)	1.6 (0.5)

*dB* decibels; *IOP* intraocular pressure; *OSS* OMNI Surgical System; *SD* standard deviation; *PXG* pseudoexfoliation glaucoma; *PG* pigmentary glaucoma

**Fig. 1:**
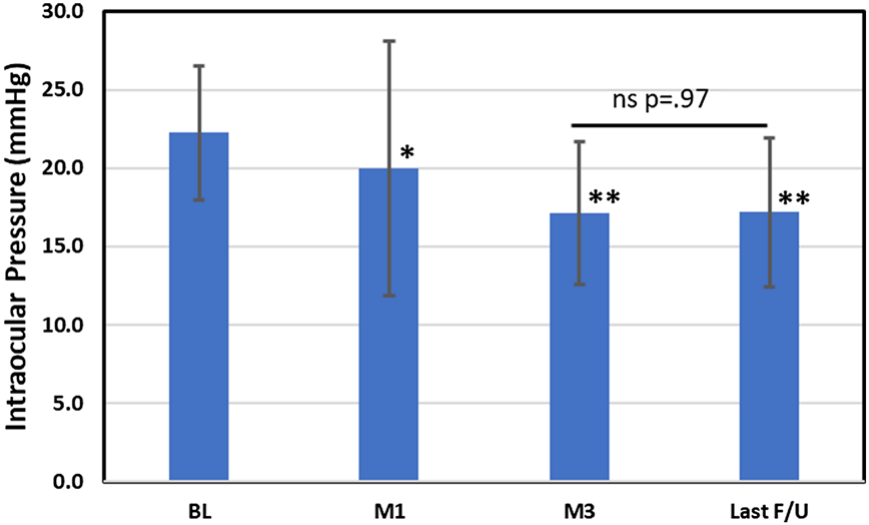
Intraocular pressure (IOP) at Baseline and Follow-up Assessments. Error bars are ± 1 SD Comparison of follow-up with baseline (Mann–Whitney rank sum): one asterisk, *p* < .01; two asterisks, *p* < .001; ns, not significant. BL = preoperative baseline, M1 = month 1, M3 = month 3, F/U = follow-up

Mean IOP was 22.3 (4.3) mmHg at BL, 17.1 (4.6) at 3 months and 17.2 (4.7) at last follow-up; with IOP reductions of 5.2 (− 23.3%) and 5.1 (− 22.9%) mmHg, respectively. IOP for each follow-up time point is presented in Fig. 1 and Table 2. Most patients had IOP at last follow-up below 21 mmHg (85%) and the majority were ≤ 18 mmHg (56%; range 8–28 mmHg). 41% had a 20% or greater reduction in IOP from the pre-OSS BL without the need for laser or additional surgery. Results for POAG and SOAG subgroups are presented in Table 2.

**Table 2:** Intraocular pressure (IOP) and medication outcomes at each post-OSS study time point

	Baseline^a^ *N* = 27	Month 1 *N* = 26	Month 3* N* = 20	Last Follow-up^b^ *N* = 27
*Mean (SD) IOP, mmHg*				
ALL	22.3 (4.3)	20.0 (8.1)	17.1 (4.6)	17.2 (4.7)
POAG	21.7 (3.1)	20.7 (8.7)	17.3 (4.5)	15.5 (3.3)
SOAG	24.7 (7.6)	16.8 (4.0)	17.1 (4.8)	17.1 (4.8)
*Eyes with decrease in IOP ≥ 20%, n (%)*				
ALL	NA	9 (35)	10 (50)	11 (41)
POAG		7 (33)	6 (40)	7 (32)
SOAG		2 (40)	4 (80)	4 (80)
*IOP ≥ 6, ≤ 18 mmHg, n (%)*				
ALL	4 (15)	11 (42)	11 (55)	15 (56)
POAG	3 (14)	9 (43)	8 (53)	12 (55)
SOAG	1 (20)	2 (40)	3 (60)	3 (60)
*Mean (SD) ocular hypotensive medications*				
ALL	2.2 (1.3)	1.7 (1.5)	1.2 (1.4)	1.8 (1.7)
POAG	2.4 (1.3)	1.9 (1.6)	1.3 (1.5)	1.8 (1.7)
SOAG	1.6 (0.5)	1.0 (1.0)	0.8 (1.1)	0.5 (1.0)
*Medication -free, n (%)*				
ALL	0 (0)	8 (31)	8 (40)	8 (30)
POAG	0 (0)	6 (29)	5 (33)	5 (23)
SOAG	0 (0)	2 (40)	3 (60)	3 (60)
*Reduction in medications ≥ 1, n (%)*				
ALL	NA	10 (37)	11 (55)	12 (44)
POAG		8 (36)	8 (53)	9 (41)
SOAG		2 (40)	3 (60)	3 (60)

*SD* standard deviation; *POAG* primary open-angle glaucoma; *SOAG* secondary open-angle glaucoma including pseudoexfoliation and pigmentary

^a^Baseline is the pre-OMNI Surgical System (OSS) baseline

^b^Last-follow-up was mean of 11.0 months post-OSS, maximum 41.2 months, minimum 3 months

**Fig. 2:**
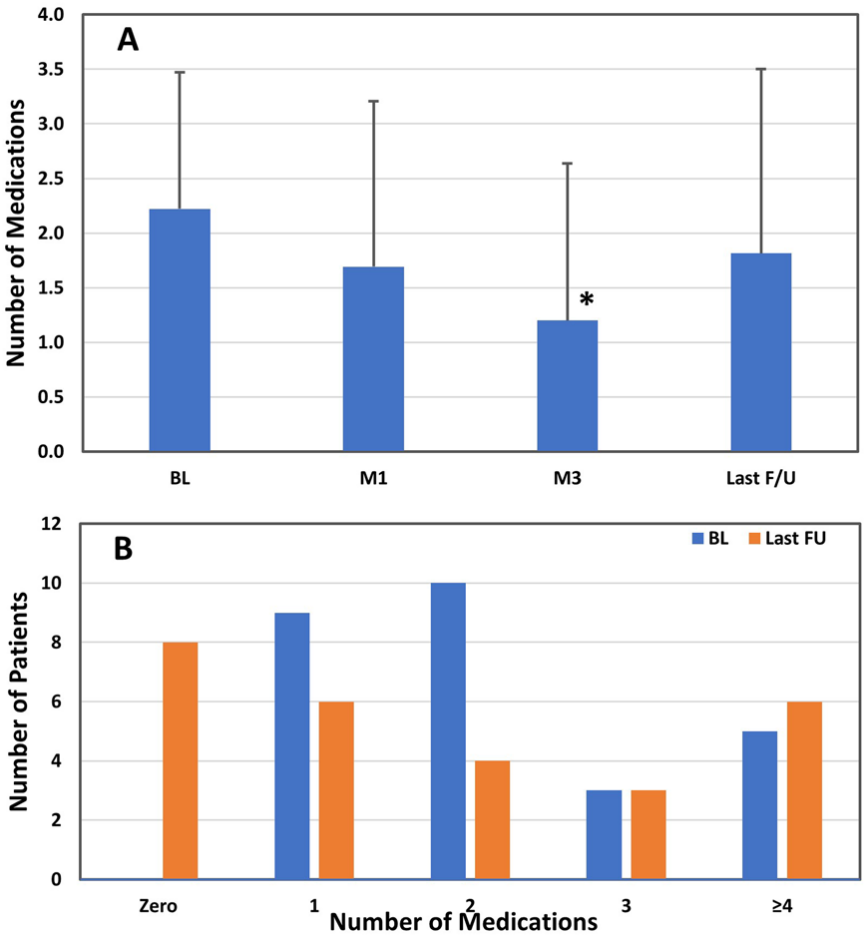
Ocular hypotensive medications. **A** Mean medications per patient at each visit. Comparison of follow-up with baseline (Mann–Whitney rank sum): one asterisk, *p* < .01. **B** Number of patients on zero, 1, 2, 3, and ≥ 4 medications at baseline and at last follow-up. BL = preoperative baseline, M1 = month 1, M3 = month 3, F/U = follow-up

Patients were under treatment with an average of 2.2 (1.3) medications at pre-OSS BL which was reduced by 1.0 and 0.4 to 1.2 (1.4) and 1.8 (1.7) medications at 3 months and last follow-up (*p* < .01 and *p* = .193, respectively) (Fig. 2A). Most (81.5%) patients were on the same or fewer medications at last follow-up as at the pre-OSS BL; 12 had a decrease in medications, 5 an increase, and 10 had the same number. Moreover, no patients were untreated with medication at BL, while 8 were (30%) at last follow-up (Fig. 2B).

IOP and medication reductions were similar between the 7 patients with the shortest follow-up (mean = 96 days) and the 7 with the longest (mean = 770 days). These were 5.3 mmHg and 5.7 mmHg, respectively; both subgroups had a 0.7 medication reduction. When the last follow-up for each patient is grouped into 3, 6, 12, and ≥ 24 month categories, change in IOP was − 4.7 mmHg at 3 months and − 6.0 mmHg at 24 months or more (Table 3).

**Table 3:** Intraocular pressure and medication use grouped by timing of last follow-up

	Baseline *n* = 27	1 month *n* = 26	3 months *n* = 20	6 months *n* = 6	12 months *n* = 6	≥ 24 months *n* = 5
Mean IOP change (SD)	22.3 (4.3)	− 2.3 (8.4)	− 4.7 (5.5)	− 6.8 (5.7)	− 3.2 (2.5)	− 6.0 (7.1)
Medications	2.2 (1.3)	1.7 (1.5)	1.2 (1.4)	2.5 (1.9)	1.5 (1.5)	1.8 (1.3)

A subgroup analysis was also carried out for patients classified by severity of glaucoma using visual field mean deviation (MD). MD >  − 6 dB was considered early, between − 6 and − 12 dB moderate, and <  − 12 dB severe. There were 12 early, mean (SD) MD 0.4 (2.58) dB, 8 moderate − 8.2 (1.66) dB, and 7 advanced − 15.3 (2.61) dB. Two of the SSI occurred in the early subgroup and two in the advanced. Mean IOP at baseline was 20.8 (3.8), 23.8 (5.8), and 23.0 (2.6) mmHg for early, moderate, and advanced, respectively. At last follow-up these were 18.3, 15.8, and 14.7 mmHg representing average IOP reductions of 5.5, 8.0, and 8.0 mmHg. The proportion of patients with a 20% or greater IOP reduction at last follow-up was 17% (early; median follow-up 176 days), 75% (moderate; median follow-up 216 days), and 71% (advanced; median follow-up 268 days).

**Fig. 3:**
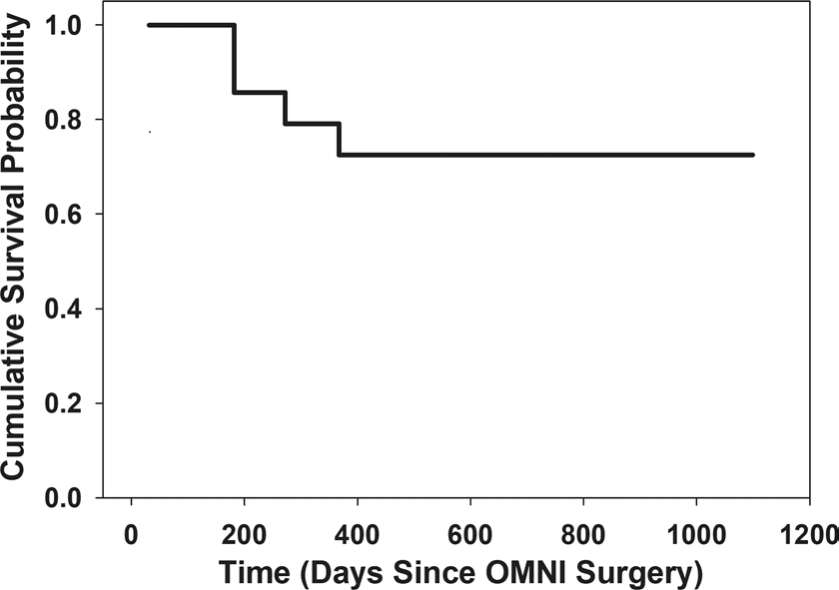
Kaplan–Meier estimates of survivor function (no secondary surgical intervention) for the 27 study patients

The OSS procedure was well tolerated. Adverse events were all non-serious and generally transient and self-resolving. The most common adverse events were 2 line (Snellen) BCVA decrease (4, 15%) which in both instances occurred at the last follow-up, clinically significant hyphema > 1 mm (3, 11%) and IOP elevation ≥ 10 mmHg above baseline (2, 7.4%). There were four patients that required an SSI (15%). Two SLTs at 3- and 5-months post-OSS (treating the hemisphere that had not undergone trabeculotomy), a XEN gel stent at 10.6 months, and an Ahmed valve at 8 months. All SSI occurred within the first post-surgical year. Cumulative probability of survival (remaining SSI-free) is depicted in a Kaplan–Meier plot (Fig. 3).

## Discussion

The interval between TBS implantation and OSS averaged about 5 years. It seems reasonable to assume that most, if not all, of the TBS were implanted according to the product label and therefore patients were no worse than moderate glaucoma when implanted. Even so, at least one quarter did progress to advanced glaucoma (− 12.01 to − 20.00 dB MD) over this interval. Moreover, by the time of the decision for OSS, average medicated IOP was 22.3 mmHg despite two-thirds of patients being on 2 or more medications and 30% on 3 or more medications.

Glaucoma is a disease marked by progressive deterioration of visual function resulting from loss of retinal ganglion cells [9]. The rate of progression is dependent on individual characteristics and risk factors which include age, degree of existing glaucomatous damage, type of glaucoma (e.g., pseudoexfoliative), prior glaucoma surgery, and peak IOP [10]. The most important goal of treatment is to preserve vision (i.e., limit or halt progression), and in a way that does not create problems worse than the disease itself [11]. Reduction of IOP to a pressure consistent with visual field stability or at least to limit visual field loss such that it is unlikely to substantially affect a patient’s health-related quality of life over the patient’s lifetime is the key goal of treatment [12]. Treatment escalation is often necessary to achieve this goal and has been shown to reduce the observed rate of disease progression [13]. For many patients, particularly with early glaucoma, escalation of treatment means the addition of another class or classes of topical IOP-lowering eyedrops. While the IOP response to the initial first medication is generally good, e.g., > 20% IOP reduction, the IOP-lowering effect diminishes with 2nd, 3rd, and 4th drops to about 4% each [14]. Increasing the complexity of drop therapy is also known to decrease patient adherence [15, 16], which could contribute to the diminishing overall effectiveness, and increase undesirable effects on the ocular surface [17]. Alternatively, MIGS, particularly when there is comorbid cataract, is increasingly being utilized in mild to moderate glaucoma for patients inadequately controlled on IOP-lowering drops, or where there may be tolerance or adherence issues [1, 2]. TBS devices are frequently used as a first MIGS procedure perhaps in part due to ease of device use and a desire to minimally disrupt or alter outflow anatomy. Current labeling limits US usage (but not in other jurisdictions, e.g. Europe) of TBS to mild-to moderate POAG with implantation concomitant with cataract surgery and reflects the TBS experience for the patients considered in the current study. But what is a reasonable next step once a TBS and additional medication are no longer sufficient to maintain IOP as desired? The present study shows that for many of these patients good IOP control can be achieved with a second MIGS procedure, in this study, canaloplasty and trabeculotomy with the OSS. Over a follow-up period that averaged close to 1 year, mean IOP was returned to the mid-teens with a modest albeit not statistically significant decrease in average medication use. Where no patients were medication-free prior to OSS, nearly one-third were following OSS.

Several studies have been published evaluating the effectiveness of the OSS in mild-moderate glaucoma as a standalone procedure [18, 19] and in combination with phacoemulsification cataract surgery [20, 21]. As a standalone procedure, Vold et al. demonstrated meaningful IOP and medication reductions for patients requiring IOP reduction, i.e., where baseline medicated IOP exceeded 18 mmHg, of 6.2 mmHg and 0.5 medications at 12 months. In a single-center study Klabe observed IOP reductions of 10–12 mmHg from a washed-out baseline through 24 months [19]. When combined with cataract surgery, a retrospective study found OSS treatment resulted in an average medicated IOP reduction of 6.9 mmHg and a 0.9 medication reduction at 12 months [20]. Patients in the prospective GEMINI study, employing a preoperative medication washout, had an average 8.2 mmHg IOP reduction at month 12 while reducing medications from 1.8 to 0.4 [21]. Moreover, post-hoc analysis of the GEMINI data found that diurnal fluctuations in IOP were significantly reduced [22].

The results of the present study are comparable with other studies of various MIGS used to treat poorly controlled glaucoma. Sarkisian et al. treated a series of patients with refractory OAG with 360 degree ab-interno trabeculotomy using the TRAB360 device [23]. The mean reduction in IOP at 12 months was 7.3 mmHg, a greater reduction than we report here, however baseline IOP was 1.4 mmHg greater in that study and 40% of the patients were surgically naïve. Medications were reduced from 1.7 to 1.1. Secondary surgical intervention was required for 25% of the patients in that series. Garcia-Feijoo et al. evaluated the CyPass supraciliary microstent in 65 patients with mean baseline medicated IOP of 24.5 mmHg on an average 2.2 medications which was reduced to 16.4 mmHg and 1.4 medications at 12 months [24]. All patients were surgically naïve. At the end of the 1-year follow-up period 17% of the eyes had undergone a secondary glaucoma surgery. In another study of the CyPass microstent, Kerr et al. implanted 20 refractory eyes, all of which had failed prior filtration surgery [25]. Baseline IOP was very similar to the present study at 22.5 mmHg although medication usage was somewhat greater (2.7 versus 2.2 medications). After one year, mean IOP was 14.9 mmHg on an average 1.2 medications. Additional glaucoma surgery was needed for 2 (10%) patients.

While MIGS are generally considered to be best suited to mild-moderate glaucoma, and TBS are restricted by the approved US label to use with cataract surgery, it is clear from the present study and the reports reviewed above that 1) they can successfully treat many patients with uncontrolled glaucoma, and 2) treatment escalation beyond a first MIGS procedure (e.g., TBS) does not preclude a second MIGS procedure (e.g., OSS) from being used successfully. The choice of OSS as a next step in these patients is reasonable given that it is a combination of two distinct procedures, canaloplasty, which addresses distal resistance in the conventional outflow pathway (Schlemm’s canal and the collector channels), and trabeculotomy which addresses resistance residing in the trabecular meshwork and inner wall of Schlemm’s canal. Importantly, the outflow system is treated circumferentially (canaloplasty) or hemi-circumferentially to circumferentially (trabeculotomy) rather than focally or for a limited number of clock hours. In an anterior segment perfusion model using cadaver eyes, Toris et al. demonstrated that the improvement in outflow facility achieved was directly related to the number of clock hours treated and to the size of the inlet (or bypass). Outflow facility improvement relative to a sham procedure was Hydrus > 2 1st generation iStents > 1 1st generation iStent > 2 iStent inject [26]. While circumferential treatment with canaloplasty and trabeculotomy was not evaluated, it can be speculated that the additional clock hours treated would potentially access more collector channels and further increase facility.

The sequence of interventions in the glaucoma treatment algorithm has undergone substantial evolution over the past decade and a half. Where once patients uncontrolled by medical therapy were destined for trabeculectomy or a tube shunt, there are now minimally invasive options that can be employed reserving traditional surgeries for severe glaucoma where a very low target IOP is required [27]. The OSS is one such option which has US FDA clearance for IOP reduction in adult phakic or pseudophakic patients with POAG. The findings of the present study extend this evolution showing that a minimally invasive option is a reasonable choice even after a first MIGS procedure with cataract surgery.

Our study has limitations. First, the study consists of a relatively small number of patients. This may, at first, seem surprising given that 5 centers contributed patients implanted with a TBS over a several year period. But considering that eligibility was limited to those eyes considered uncontrolled by medication and where the TBS was no longer providing the desired degree of control (IOP > 17 mmHg and on at least 1 medication), eyes that remained at 17 or below, or medication-free were not included. Moreover, to avoid confounding influence of other procedures, any eyes that had received a different surgical or laser procedure following TBS and before OSS could not be included. Our findings are overall consistent with other published evaluations of MIGS in medically uncontrolled glaucoma. Moreover, the study population consists of patients from several centers and surgeons mitigating the potential for bias that can be associated with single-center studies. Second, the minimum follow-up time of 3 months could be seen as limiting the utility of the study outcomes for predicting long-term success or failure of the OMNI procedure in similar patients. However, one quarter of the patients had greater than 1 year of follow-up and when this quartile was compared with the quartile with the shortest follow-up, the IOP and medication reductions were very similar supporting the reliability of the overall outcomes reported and the durability of the treatment effect. In addition, all instances of required secondary surgical intervention occurred within the first year, the last was at 322 days. Third, this was a retrospective study without a comparator. However, all available eligible cases were included, and exclusion criteria were kept to a minimum. Lastly, while we have discussed the relative severity of glaucoma for the included patients for informational purposes, we acknowledge the potential inaccuracy of staging based solely on visual field mean deviation from single visual fields.

## Conclusion

It is our belief that the data from this study closely mirrors outcomes for similar patients treated by other surgeons. We believe that additional study of OSS in this population is warranted and look forward to confirmation of our results in additional studies by other investigators.
